# Odd haemoglobins in odd-toed ungulates: Impact of selected haemoglobin characteristics of the white rhinoceros (*Ceratotherium simum*) on the monitoring of the arterial oxygen saturation of haemoglobin

**DOI:** 10.1371/journal.pone.0226851

**Published:** 2019-12-30

**Authors:** Julia K. Reiners, Nadja Hellmann, Juliane Schmidt, Sabine B. R. Kästner

**Affiliations:** 1 Department of Anaesthesiology, University of Veterinary Medicine Hanover, Foundation, Hanover, Germany; 2 Institute for Molecular Biophysics, University of Mainz, Mainz, Germany; Boston University, UNITED STATES

## Abstract

**Background:**

Due to the current poaching crisis in Africa, increasing numbers of white rhinoceroses (*Ceratotherium simum*) require opioid immobilisation for medical interventions or management procedures. Alarmingly, the results of both blood gas analysis and pulse oximetry regularly indicate severe hypoxaemia. Yet, the recovery of the animals is uneventful. Thus, neither of the techniques seems to represent the real oxygenation level. We hypothesized that unusual haemoglobin characteristics of this species interfere with the techniques developed and calibrated for the use in human patients.

**Methods:**

Haemoglobin was isolated from blood samples of four adult, white rhinoceroses. Oxygen dissociation curves at pH 7.2 and 7.4 (37°C) were determined based on the absorbance change of haemoglobin in the Soret-region (around 420 nm). Absorbance spectra of oxy- and deoxyhaemoglobin extending into the infrared region were measured.

**Results:**

Oxygen dissociation curves of rhinoceros haemoglobin showed the typical high oxygen affinity (p_50_ of 2.75 ± 0.07 and 2.00 ± 0.04 kPa for pH 7.2 and 7.4, respectively) under near-physiological conditions with respect to pH, temperature and DPG. The infrared absorbance spectra of oxy- and deoxyhaemoglobin showed only marginal deviations from standard human spectra, possibly due to the presence of a few percent of methaemoglobin *in vitro*.

**Conclusions:**

Our data enables the development of a rhinoceros-specific blood gas analysis algorithm, which allows for species-specific calculation of SaO_2_ levels in anaesthetized animals. The inconspicuous absorbance spectra do not contribute to the systematic underestimation of SpO_2_ by pulse-oximetry.

## Introduction

The white rhinoceros (*Ceraotherium simum*) has been the subject of numerous publications in the field of veterinary anaesthesia in the past few years. While medical training in animals under human care allows for minor procedures like blood sampling to be performed without sedation, more invasive medical interventions and management procedures (e.g. wound treatment, dehorning, relocations) generally require so-called chemical immobilisation.

Furthermore, the current rhinoceros poaching crisis in Africa requires intense management of the populations, emphasizing the need for safe and reliable anaesthetic protocols and monitoring techniques. As standard protocols include highly potent opioids, the monitoring of the arterial oxygen saturation of haemoglobin is of paramount importance. Severe side effects (e.g. muscle tremors, tachycardia, hypertension, hypercapnia, low partial pressure of oxygen (pO_2_) and acidosis) occur regularly and are well described [1].

Under field conditions, blood gas analysis (providing calculated SaO_2_) as well as pulse oximetry (providing SpO_2_) are being used. Both monitoring techniques were developed for the use in human patients and have been implemented in veterinary anaesthesia without further adaptation of the algorithms and calibration data. In the case of certain domestic species (namely dog, cat, horse, cow and pig) the infrared spectra were shown to be indeed sufficiently close to justify this approach [2,3] for pulse oximetry. However, limited information is available for large herbivores, as the species-specific absorbance characteristics of oxyhaemoglobin and deoxyhaemoglobin in the white rhinoceros have not been studied before.

Standard blood gas analysis and pulse oximetry indicate an alarmingly low oxygenation status under opioid immobilisation that is in conflict with uneventful recoveries [1,4–6]. Haymerle et al. reported SaO_2_ values as low as 39% and SpO_2_ values as low as 42% in clinically healthy, opioid-immobilised animals generated by commercially available devices [7]. Baumann et al. presented the p50 and Hill coefficient determined for white rhinoceros haemoglobin (based on measurements on one blood sample from a single white rhinoceros) and showed that its oxygen binding properties are modulated by pH and CO_2_ but not by DPG [8]. When Haymerle et al. modified the algorithm developed by Siggaard-Andersen et al. [9] to yield the p50 value and Hill coefficient provided by Baumann and et al., SaO_2_ levels of at least 80% were calculated. Since only p50 and Hill coefficients are available from Baumann et al., we felt that the data analysis could be further improved by modifying the analysis algorithm based on more detailed experimental data, which led us to perform the corresponding experiments.

We hypothesized that deviating haemoglobin characteristics may interfere with the monitoring techniques developed and calibrated for the use in human patients. Possible sources of error include 1) deviating oxygen binding properties of haemoglobin of the white rhinoceros that interfere with the calculation of SaO_2_ by blood gas analysers using human or equine algorithms and 2) deviating light absorbance characteristics of the haemoglobins interfering with pulse oximetry as described in human patients with haemoglobinopathies [10].

Our objectives were 1) to provide oxygen dissociation curves of the white rhinoceros to allow for proper species-specific calibration of blood gas analysers and 2) to investigate the haemoglobin infrared absorbance characteristics of oxy- and deoxyhaemoglobin to check for possible deviations in the extinction coefficients relevant to pulse-oximetry.

## Methods

EDTA blood samples (5ml each) of four adult white rhinoceroses (one male, three females; aged seven to twenty-six years) housed in European zoological institutions were used for this study. All of these Institutions are members of the *European Association of Zoos and Aquaria* (EAZA) and participate in the *European Endangered Species program* (EEP). The samples were taken between June 2014 and December 2018. All animals were considered healthy based on the clinical assessment of the veterinary clinician in charge. All samples were collected during routine blood sampling for health monitoring and leftover specimens were secondarily donated to our study. Therefore, the obtainment of ethical approval was not required.

Equine blood samples were also examined. In exotic species, clinically relevant basic information is often rare; therefore, it is common practise to consult the literature on closely related domestic animals for approximation and comparison. As the haemoglobin characteristics of the domestic horse (*Equus caballus*) had been described before [3], we decided to include both members of the order *Perrisodactyla* into our investigations for internal comparison.

We used blood samples from two clinically healthy warmblood horses (one gelding, one mare; two and twelve years old) that were experimental horses in the possession of the equine clinic at the University of Veterinary Medicine Hanover. For the blood sampling of the horses, ethical approval was granted by the Ethics Committee for the Animal Experiments of Lower Saxony (Lower Saxony State Office for Consumer Protection and Food Safety, approval number 33.19-42502-04-18/2856). The skin over the left jugular vein was clipped and surgically prepared for catheter placement. After infiltration of the skin with mepivacaine hydrochloride (Scandicain 2%, AstraZeneca, Wedal, Germany), a 12 G catheter (EquiCath Fastflow, Braun, Tuttlingen, Germany) was placed into the left jugular vein. Blood was drawn from the catheter aseptically. The first 10 ml were discarded, then the sample (approximately 10 ml) was withdrawn and placed in EDTA tubes.

### 1. Determination of the oxygen dissociation curve (ODC)

#### 1.1. Chemicals

Buffer components (TRIS, NaCl) were obtained from Roth (Roth Chemicals, Karlsruhe, Germany). All components of the Hayashi assay and Sodium Dithionite were purchased from Sigma (now Merck KGaA, Darmstadt, Germany). Buffer for oxygen binding experiments contained 0.05 M TRIS/HCl with a concentration of chloride of 0.1 M adjusted with NaCl.

#### 1.2. Oxygen dissociation curves (ODC)

Haemoglobin was isolated from secondarily donated samples from rhinoceroses housed around Europe. Therefore, pre-analytic handling of the samples (including shipping, preparation and storage) was necessary. Upon arrival at the laboratory, haemoglobin was extracted from the blood sample, using the method described by Paoli und Nagai [11]: first, the blood was centrifuged for 30 min at 100 g (4°C). Then the supernatant was removed, the pellet carefully mixed with at least 10 times the volume of 0.9% NaCl and centrifuged again (30 min, 100 g, 4°C). The supernatant was removed and the procedure repeated until the supernatant was clear. Then the erythrocytes were lysed by addition of 1x volume of water. After 15 min, 9% NaCl was added to obtain a final concentration of about 5% (w/v) NaCl. Next, the cellular debris was removed by centrifugation (4100 g, 30 min, 4°C). The supernatant contained the haemoglobin. The purified haemoglobin was stored at 4°C. The procedure was performed on the day of arrival of the blood sample. Isolated oxygenated haemoglobin can be stored at 4°C for at least two months [12]. Samples were used within this time period and always checked for increased met-formation beforehand. For increasing concentration or buffer exchange, centrifugal concentrators were used (Centricons Vivaspin 20 ml, 30.000 Da, Sartorius Stedim Biotech GmbH, Göttingen).

Oxygen dissociation curves were measured in a Gill-cell [13] with an optical path length of about 0.05 mm. In order to prevent formation of methaemoglobin during the measurement, the regeneration assay after Hayashi [14] was included into the experiments. ODCs were performed with a solution of the following composition: haemoglobin at a concentration of about 200 μM haemoglobin tetramer, 0.04 μM catalase (about 6 U/ml), 0.5 μM ferredoxin, 0.27 U/ml Glucose-6-phosphate-dehydrogenase, 0.15 mM NADP, 3 mM Glucose-6-phosphate, 0.15 μM ferredoxin NADP^+^ reductase (about 0.1 U/ml). The solution was pre-incubated at 37° C to convert all methaemoglobin into oxyhaemoglobin, since some degree of methaemoglobin formation had already occurred during shipping of the blood sample. Prior to the measurement, the sample was shortly spun in a table centrifuge to remove air bubbles. Gas of defined mixing ratios of 20% O_2_ and 100% N_2_ (Linde Group, Pullach, Germany) was prepared in a self-built gas mixing system and led into the chamber next to the semipermeable membrane (Model 5794, High Sensitivity) from YSI Incorporated (Yellow Springs, Ohio, USA). The actual pO2 was measured in the gas chamber with an oxygen electrode (MicroEletrodes Inc., Bedford, New Hampshire, USA) with a self-built amplifier. Measurements were performed under continuous gas flow. Calibration was performed with gas of a known composition of N_2_ and O_2_ (Linde Group, Pullach, Germany). Oxygen partial pressure (pO_2_) was increased gradually. A spectrum was measured after the electrode voltage and absorption values were stable. The fraction of oxygenated protein (f_oxy_) was calculated based on a superposition of the spectra obtained for the oxygenated and the deoxygenated sample including a variable small offset:

$$S_{pO_{2}}=a_{oxy}\;S_{oxy}+a_{deoxy}S_{deoxy}+off$$

Here S_oxy_ and S_deoxy_ refer to the spectra of oxy- and deoxyhaemoglobin measured for the respective set of data, and a_oxy_ and a_deoxy_ are parameters adjusted by the fitting routine to obtain the best agreement between measured and calculated spectrum. In order to allow for baseline drifts, a constant offset („off“) was also included. The fraction of oxygenated haemoglobin was then calculated as

$$f_{oxy}=a_{oxy}/(a_{oxy}+a_{deoxy})$$

The pO_2_ was calculated from the voltage output of the Clark-electrode, taking into account the water vapor pressure (6.27 kPa at 37°C) as well as the actual ambient pressure.

### 2. Infrared absorption spectra of oxygenated and deoxygenated haemoglobin

Spectra of the undiluted haemoglobin solutions in 5% NaCl were measured employing a Lambda 465 (Perkin Elmer Inc., Waltham, Massachusetts, USA) with a cuvette of 1 cm pathlength. Deoxy-haemoglobin was prepared by adding Sodium-Dithionite to the stock solution, about 20 μl of a 10 mg/ml solution in H_2_0 for 1 ml haemoglobin solution. For comparison, spectra of equine haemoglobin were also recorded under the same conditions. In order to allow a comparison of the spectral shapes, they were normalized to the value measured at 940 nm (oxy-spectra) and to 660 nm (deoxy-spectra).

## Results

### 1. Oxygen dissociation curves

**Fig 1:**
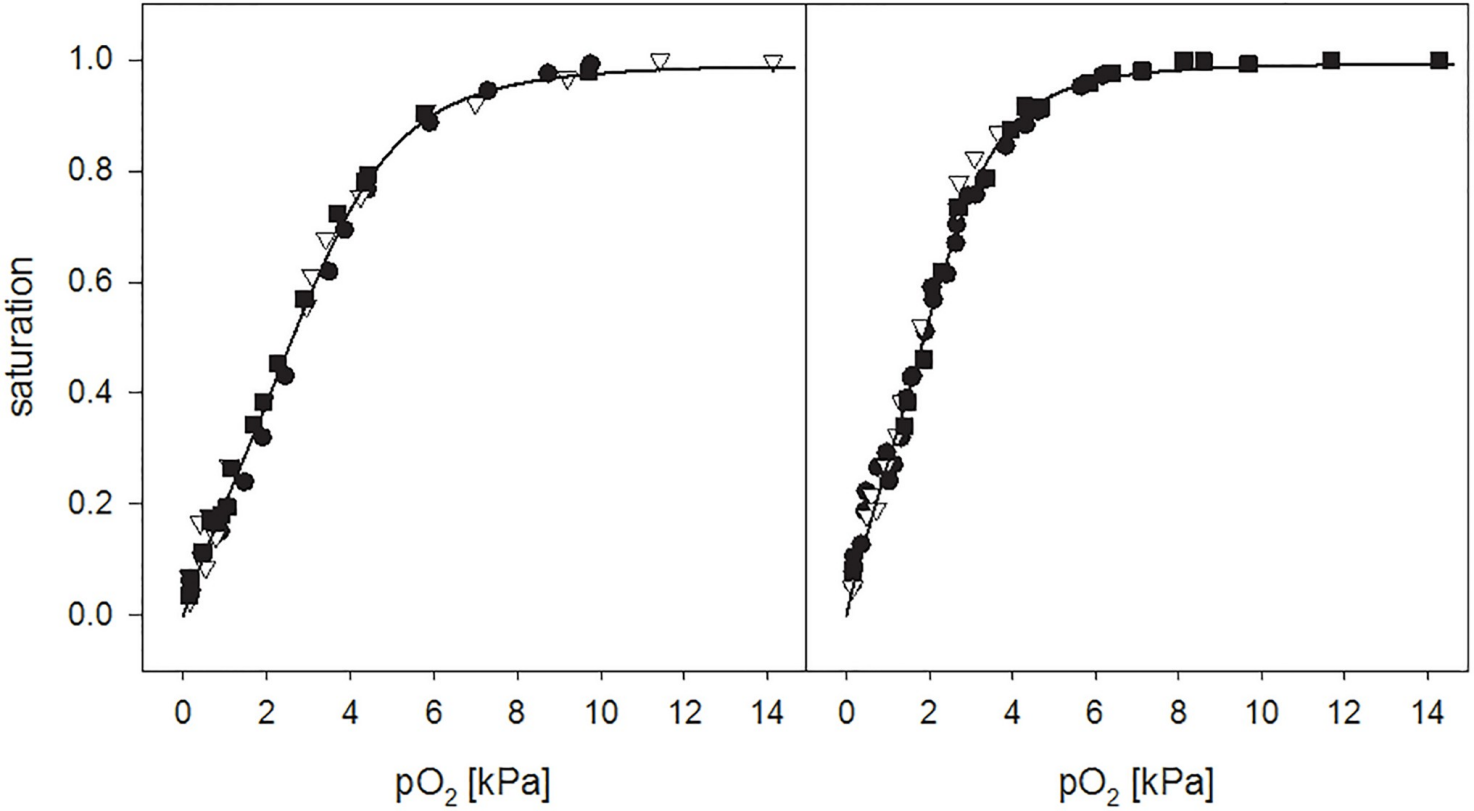
Oxygen dissociation curves of white rhinoceros haemoglobin at 37°C. ODCs were measured at pH 7.2 (left panel) and pH 7.4 (right panel) in 50 mM TRIS/HCl at 0.1 M chloride. Measurements were performed with haemoglobin isolated from three different animals, indicated by the different symbols. The data does not indicate variations in oxygen affinity for different individuals. The solid lines represent the fit based on the function described in the supplemental material (eq.1), corresponding to a modified version of the one employed for human haemoglobin for the determination of SaO_2_ from blood gas analysis.

Oxygen dissociation curves were measured at 37°C, at pH 7.2 and pH 7.4, using haemoglobin from three individuals. All data points lie on a common curve (Fig 1). For comparison with available data [8], p50 and Hill coefficient n50 were obtained based on the Hill plot of part of the data (25–80% saturation), yielding values of 2.5 and 1.78 kPa, and n50 = 2.1 and 2.2 for pH 7.2 and 7.4, respectively. The Bohr coefficient calculated from the shift in p50 amounts to -0.74, which is slightly higher than the one reported by Baumann et al. (-0.62, [8]). The p50 values reported by these authors were 2.29 kPa at pH 7.2 and 1.48 kPa at pH 7.5, being in reasonable agreement with the ones reported here. The Hill coefficients were somewhat higher, ranging from 2.8 to 2.6 between pH 7.0 and 7.5. An analysis of all data by non-linear regression based on the Hill equation yielded the following parameters for pH 7.2 and 7.4: p_50_ = 2.75 ± 0.07 and 2.00 ± 0.04 kPa, n_50_ = 2.0 ± 0.2 and 2.2 ± 0.1, respectively.

### 2. Haemoglobin absorbance spectra

**Fig 2:**
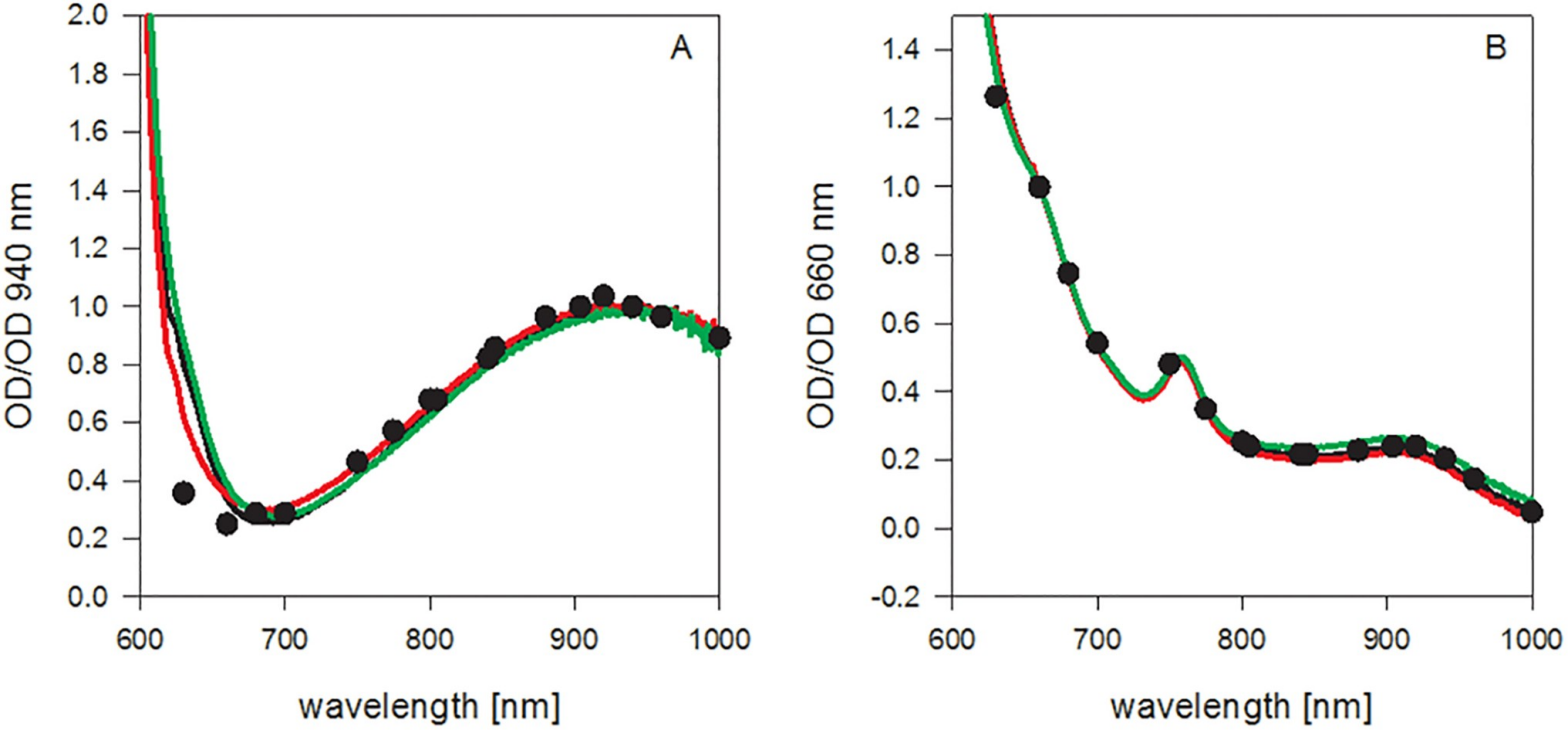
Absorption spectra of oxygenated (A) and deoxygenated (B) haemoglobin from rhinoceros (two individuals, black and red line) and horse (one individual, green line). For comparison, the spectrum of human haemoglobin, taken from Zijlstra et al. is also shown (circles). The Spectra are normalized to OD at 940 nm (panel A), and to 660 nm (panel B) to allow comparison of the spectral shape. The absorbance values were about 0.2 at 940 nm for oxyhaemoglobin and about 0.47 at 660 nm for deoxyhaemoglobin.

The haemoglobin absorbance spectra of oxyhaemoglobin and deoxyhaemoglobin of horse and rhinoceros are presented in Fig 2. No significant differences in the spectral features could be observed between the haemoglobins of human, horse and rhinoceros. The slightly enhanced absorbance in the lower wavelength range in case of the horse and rhinoceros spectra can be attributed to formation of methaemoglobin. While the Hayashi assay successfully removed methaemoglobin from the solution used for measuring the oxygen dissociation curves, it did apparently not work equally well with the high haemoglobin concentrations necessary for the measurement of the absorbance spectra.

## Discussion

As expected, the ODC of the white rhinoceros showed a marked left shift at both pH = 7.4 and pH = 7.2 compared to the human ODC under near-physiological conditions. The main effector responsible is DPG, which significantly lowers the oxygen affinity of human haemoglobin, but has no effect on white rhinoceros haemoglobin [8]. However, the absorbance spectra of oxy-and deoxyhaemoglobin in both white rhinoceros and domestic horse showed only minor deviations from human absorbance patterns, most likely due to the presence of residual methaemoglobin *in vitro*.

Based on the two ODCs presented, mathematical models like the one established by Siggaard-Andersen et al. [9] and modified by Haymerle et al. [7] can now be fitted directly to measured curves, yielding rhinoceros-specific parameters (Tab A in S1 Appendix). These species-specific parameters are valid only under the specified measuring conditions (pH, temperature, salt concentration). The effect of further modulators such as CO_2_ on the p50 can be incorporated as demonstrated by Haymerle et al. ([7], see S2 Fig and Tab B in S1 Appendix), Thus, SaO_2_ can now be estimated based on pO_2_, pCO_2_ and pH determined through blood gas analysis. To demonstrate this, we used the experimental values reported by Haymerle et al. [7] and combined them with our parameters to calculate SaO_2_, resulting in saturation levels above 80% in most cases (S2 Fig). Arterial haemoglobin saturation values below 90% still indicate hypoxaemia, but saturation levels above 80% seem more plausible than values of less than 40% as reported in the literature.

Acidosis, hypercapnia and a low arterial partial pressure of oxygen (PaO_2_) are common in opioid-immobilised white rhinoceroses, indicating marked cardiorespiratory depression [1,4–6]. Our findings help to explain why despite the seemingly extremely low SaO_2_ values reported in these animals, recovery is usually uneventful, without obvious clinical signs of long-term damage associated with such severe hypoxaemia. As stated above, some level of hypoxaemia is still very likely to occur in immobilised white rhinoceroses, underlining the importance of adequate anaesthetic protocols and management by trained professionals.

It should be kept in mind that the model is based on ODCs measured *in vitro*; we cannot exclude that other, so far unknown factors are present under physiological conditions *in vivo*, having additional effects on SaO_2_ [6]. The presented ODCs can now be used to construct a model to estimate SaO_2_ more reliably, allowing for a better understanding of the true oxygen saturation status of white rhinoceroses during opioid immobilisation. Meanwhile, tabulated saturation levels at both pH 7.2 and pH 7.4 (Tab C in S1 Appendix) can aid colleagues in the field to interpret the results of blood gas analysis.

A high oxygen affinity of haemoglobin under in vivo-like conditions (characterised by a left-shifted ODC) is a typical finding in large mammals, as it facilitates oxygen uptake in the lungs and its transport via blood over longer distances. However, O_2_ delivery cannot be characterized by haemoglobin’s oxygen binding characteristics only; instead, physiological determinants of oxygen transport are “redundant and numerous”, including blood flow (cardiac output), ventilation and acid-base-status [15]. Interestingly, Haymerle et al. found evidence that white rhinoceroses have a higher cardiac output than humans (expressed per square meter of body surface) [7], which could be interpreted as an evolutionary adaptation to ensure adequate O_2_ supply.

Specific mechanisms facilitate the deoxygenation of haemoglobin and therefore the final delivery of the oxygen to the target tissues, including the Bohr effect, Haldane effect and other allosteric factors. Baumann et al. reported that in the haemoglobin of the white rhinoceros, only protons and chloride anions are major allosteric factors controlling its oxygen affinity, while DPG and CO_2_ are not [8]. The Bohr and Haldane effects are closely linked and are characterised by the species-specific Bohr coefficient; values reported for the white rhinoceros (-0.62 by Baumann et al. and -0.74 in our study) are high compared to other species.

It is interesting to consider the clinical consequences of the high Bohr coefficient for the situation under immobilisation: especially under field conditions, where chasing occurs prior to darting, animals show severely elevated levels of lactate [16], indicating an anaerobic metabolic status. According to Lapennas [17], a higher Bohr coefficient is favourable under these circumstances, as it produces a right-shift of the ODC, which ensures sufficient oxygen supply of the metabolic active tissues and contributes to overcome the anaerobic situation. This information facilitates a better understanding of how white rhinoceroses are actually able to cope with the physiologically extreme situation under immobilisation with highly potent opioids.

In human patients, certain haemoglobin anomalies (Hb Bonn, Hb Cheverly, HbM Iwate, Hb Köln) are known to cause falsely low SpO_2_ readings due to deviating absorbance curves, which interfere with the calculation of SpO_2_ based on the standard 660nm/ 940nm absorbance ratio [10]. This led us to hypothesize that deviating absorbance characteristics of white rhinoceros oxy- and deoxyhaemoglobin might contribute to the low SpO_2_ readings. However, only minor deviations in the absorbance curves measured by us for horse and rhinoceros haemoglobin from the data on human haemoglobin published by Zijlstra et al. [18] were observed.

The residual methaemoglobin, which was formed during shipping and could not be removed from the sample material at the high haemoglobin concentrations needed for the measurements, could be held accountable for this deviation. Due to methaemoglobin’s extinction coefficients at the two wavelengths recorded, presence of methaemoglobin will increase the ratio measured (readings at 630 nm/readings at 940 nm), and pulse-oximetry would indeed indicate artificially decreased levels of SaO_2_.

The relevance of these findings for the situation *in vivo* remains unclear. Interestingly, due to elevated free tyrosine levels in the erythrocytes of all members of the order *Perissodactyla*, order-specific processes of free radical and antioxidant metabolism have been discussed before [19]. Indicators of oxidative stress are well described in exercising horses [20]. One could argue that an opioid immobilisation including the severe side effects resembles exercise, potentially–as it is a drug-induced and uncontrolled effect—challenging the antioxidative capacity of rhinoceros erythrocytes, leading to oxidative stress and potentially the formation of methaemoglobin *in vivo*.

In addition, the absorbance of purified haemoglobin does not necessarily represent the situation *in vivo*, where components of the erythrocytes, whole blood and surrounding tissues might interfere with the photometric measurement. Other known interference factors of pulse oximetry include excessive movement, venous pulsation, poor perfusion and poor probe positioning [21], all of which might well be relevant in the case of opioid-immobilised white rhinoceroses.

A limitation of our study is the small sample size, which is a common problem in veterinary studies on rare species. In this context it is worth mentioning that the southern white rhinoceros population experienced an extreme bottleneck effect in the late 19^th^ century with not more than 50 animals left [22], so today’s population of around 20 000 animals worldwide can be expected to have a low genetic diversity.

Hypoxaemia remains a major issue in white rhinoceroses under opioid immobilisation. The results of this study provide evidence that the arterial oxygen saturation of haemoglobin is regularly underestimated by SaO_2_ derived from blood gas analysis based on human data. Furthermore, our findings help to construct a model that estimates the oxygen saturation more accurately. However, no obvious deviation in absorbance spectra could be found *in vitro* to explain a potential underestimation of SpO_2_ by pulse oximetry. An extraction and analysis of the crystal structure of the white rhinoceros haemoglobin is in progress in order to further investigate this question.

## Supporting information

S1 Appendix Main, combined file. Supporting Information on 1. The construction of an equation describing white rhinoceros ODCs and 2. The determination of haemoglobin saturation by blood gas analysis.
(DOCX)

S1 Fig Employing eq.1 and eq.2, ODCs were calculated for different pH and different pCO_2_. The corresponding p50 values show a decreasing impact of pCO_2_ at decreasing pH-values, in agreement with the results reported by Baumann et al.
(TIF)

S2 Fig Employing the value of blood gas analysis (pH, CO_2_, pO_2_, temperature) of anaesthetized rhinoceros published by Haymerle and colleagues, the saturation level of white rhinoceros haemoglobin was estimated based on eq.1+eq.3 (black circles) or in a simplified version, where temperature, Hi and HbCO is not included (eq.1 + e.q2., red circles). (TIF)

S3 Fig Based on the function suggested by Haymerle et al., oxygen binding curves were calculated for two different pH values and two different pCO_2_ values, in the range found in the blood of anaesthetized rhinoceros. Note that an increase in pCO_2_ and a decrease in pH leads to a left-shift of the ODC, in contrast to experimental data by Baumann and colleagues. In contrast, the function represented by eq.2 and eq.3 reflect the experimentally observed shifts, since the experimental data were used to generate the equations.
(TIF)
