# Supplementary material for: Odd haemoglobins in odd-toed ungulates: Impact of selected haemoglobin characteristics of the white rhinoceros (Ceratotherium simum) on the monitoring of the arterial oxygen saturation of haemoglobin
Source: PLoS One. 2019 Dec 30;14(12):e0226851. doi: 10.1371/journal.pone.0226851 (PMC6936770; doi:10.1371/journal.pone.0226851)
Supplement: S1 Appendix — Supporting Information on 1. The construction of an equation describing white rhinoceros ODCs and 2. The determination of haemoglobin saturation by blood gas analysis. (DOCX) [file pone.0226851.s001.docx]

**Supporting Information**

**1. Construction of an equation describing white rhinoceros ODCs**

The function suggested by Siggaard-Andersen et al. in 1998 to describe the ODC of human haemoglobin was rewritten in the following version and fitted to the two ODCs shown in Fig 1:

$s=\frac{e^{y}}{1+e^{y}}$

$y=y_{s}+x-x_{s}+h tanh(k\left( x-x_{s} \right))$ eq.1

Like done for human haemoglobin, ys and k are assumed to be independent of effector concentration (CO_2_, pH). In case of white rhinoceros haemoglobin, a constant value of h was additionally assumed, since allowing h to depend on pH did not improve the fit significantly. The main consequence of effectors is to shift the position of the curve, represented by xs, which is therefore allowed to differ for the two ODCs.

| pH | 7.2 | 7.4 |
| --- | --- | --- |
| xs | 1.770 ± 0.198 | 1.431 ± 0.198 |
| h | 1.835 ± 0.462 | |
| k | 1.169 ± 0.188 | |
| ys | 2.177 ± 0.630 | |

**Tab A.** Parameters obtained by fitting eq.1 simultaneously to the two ODCs shown in Fig1.The errors are those calculated by the fitting routine.

From the shift in x_s_, which indicates the point of symmetry, an apparent Bohr coefficient of
-1.7 can be calculated. Of note: this value should not be confused with the Bohr coefficient determined from the shift in p50, which is much less (see main text).

The influence of pH on the point of symmetry of the ODC can thus be parameterised as follows, with x_o_ being the value of x_s_ at pH 7.4:

$x_{s}=x_{o}+a$ $a=-1.7\left( pH-7.4 \right)$

Baumann et al. showed that similar to human haemoglobin, presence of CO_2_ shifts the p_50_ to higher values. However, this shift does not occur at pH 7.2, only at higher pH values. At pH 7.5, the p_50_ shifted from 1.48 to 1.97 kPa, at pCO_2_ of 5.33 kPa, corresponding to a shift of log (p_50_) of 0.125. The shift in p_50_ can be reproduced if one uses the following term for the shift in x_s_ due to presence of CO_2_ and changes in pH:

$a=-1.7\left( pH-7.4 \right)+1.38 \ln\left( 1+\frac{pCO2}{5.33} \right)(pH-7.2)$ eq.2

Here, at low pH values the effect is small (see S1 Fig), vanishing at pH = 7.2. For pH values < 7.2, one just has to set pCO_2_=0 in order to represent the missing effect of CO_2_ under these conditions. Binding curves were simulated for the conditions listed in Tab A, and the corresponding p50 were retrieved:

| pH | pCO_2_ [kPa] | p50 (kPa) |
| --- | --- | --- |
| 7.4 | 0 | 1.86 |
| 7.5 | 0 | 1.57 |
| 7.5 | 5.33 | 2.10 |
| 7.2 | 0 | 2.62 |
| 7.2 | 5.33 | 2.62 |

**Tab B.** p50 values under different pH and pCO_2_ conditions in the white rhinoceros.

The shift in log p50 at pH 7.5 due to presence of 5.33 kPa CO_2_ is 0.127, in good agreement with the data from Baumann et al.. The p50-values are not exactly the same as in the experiments, differing by 0.133 kPa, but we wanted to represent the whole curve as well as possible, in particular the upper part, rather than the p_50_ itself, which is usually outside the physiological pO_2_ values.


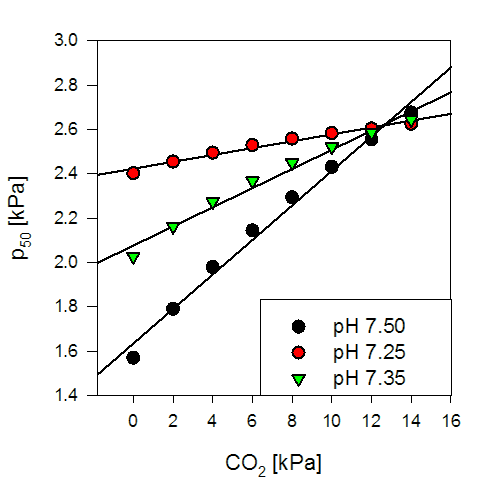


**S1 Fig.** Employing eq.1 and eq.2, ODCs were calculated for different pH and different pCO_2_. The corresponding p50 values show a decreasing impact of pCO_2_ at decreasing pH-values, in agreement with the results reported by Baumann et al..

**2. Determination of haemoglobin saturation by blood gas analysis**

Finally, in order to include the effect of T (in °C), presence of Hi and HbCO, the terms used for human haemoglobin were also included:

$x_{s}=x_{o}+a+b$ $b=0.055*(T-37^{\circ})$

$a=-1.7\left( pH-7.4 \right)+1.38\ln\left( 1+\frac{pCO2}{5.33} \right)(pH-7.2)-0.368*xHbCO-0.174xHi$ eq.3

Employing this function, the haemoglobin saturation levels based on the experimental data from blood gas analysis supplied by Haymerle et al. was estimated, as shown in S2 Fig. However, the effect of HbCo, Hi and temperature in the range as found experimentally (see Haymerle et al.) is small (compare the red and black symbols in S2 Fig) and can therefore be neglected. Thus, the simpler variant (eq.2) is sufficient to describe the oxygen binding characteristics of white rhinoceros haemoglobin.


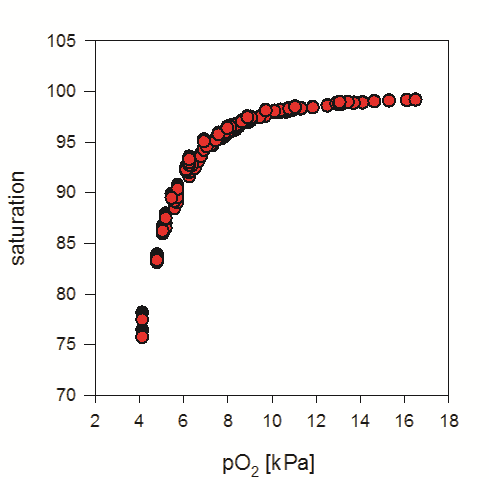


**S2 Fig.** Employing the value of blood gas analysis (pH, CO_2_, pO_2_, temperature) of anaesthetized rhinoceros published by Haymerle et al., the saturation level of white rhinoceros haemoglobin was estimated based on eq.1+eq.3 (black circles) or in a simplified version, where temperature, Hi and HbCO is not included (eq.1 + e.q2., red circles).


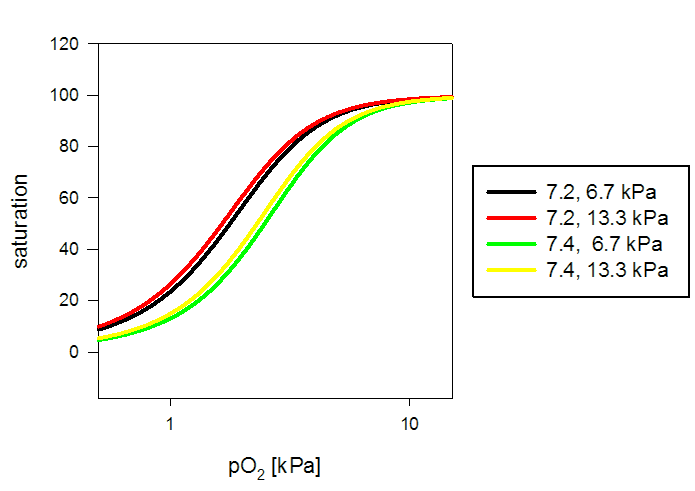


**S3 Fig.** Based on the function suggested by Haymerle et al., oxygen binding curves were calculated for two different pH values and two different pCO_2_ values, in the range found in the blood of anaesthetized rhinoceros. Note that an increase in pCO_2_ and a decrease in pH leads to a left-shift of the ODC, in contrast to experimental data by Baumann and colleagues. In contrast, the function represented by eq.2 and eq.3 reflect the experimentally observed shifts, since the experimental data were used to generate the equations.

| pO_2_ [mmHg] | pO2 [kPa] | SaO_2_, pH 7.4 | SaO_2_, pH 7.2 |
| --- | --- | --- | --- |
| 0.0 | 0 | 0.0000 | 0.0000 |
| 2.0 | 0.266 | 0.0819 | 0.0596 |
| 4.0 | 0.532 | 0.1545 | 0.1135 |
| 6.0 | 0.798 | 0.2227 | 0.1638 |
| 8.0 | 1.064 | 0.2901 | 0.2124 |
| 10.0 | 1.330 | 0.3586 | 0.2603 |
| 12.0 | 1.596 | 0.4286 | 0.3085 |
| 14.0 | 1.862 | 0.4990 | 0.3574 |
| 16.0 | 2.128 | 0.5680 | 0.4071 |
| 18.0 | 2.394 | 0.6332 | 0.4572 |
| 20.0 | 2.660 | 0.6925 | 0.5073 |
| 22.0 | 2.926 | 0.7446 | 0.5564 |
| 24.0 | 3.192 | 0.7891 | 0.6039 |
| 26.0 | 3.458 | 0.8261 | 0.6488 |
| 28.0 | 3.724 | 0.8564 | 0.6905 |
| 30.0 | 3.990 | 0.8809 | 0.7286 |
| 32.0 | 4.256 | 0.9006 | 0.7628 |
| 34.0 | 4.522 | 0.9164 | 0.7931 |
| 36.0 | 4.788 | 0.9291 | 0.8197 |
| 38.0 | 5.054 | 0.9393 | 0.8428 |
| 40.0 | 5.320 | 0.9475 | 0.8627 |
| 42.0 | 5.586 | 0.9543 | 0.8798 |
| 44.0 | 5.852 | 0.9598 | 0.8944 |
| 46.0 | 6.118 | 0.9644 | 0.9069 |
| 48.0 | 6.384 | 0.9682 | 0.9175 |
| 50.0 | 6.650 | 0.9714 | 0.9267 |
| 52.0 | 6.916 | 0.9741 | 0.9345 |
| 54.0 | 7.182 | 0.9764 | 0.9412 |
| 56.0 | 7.448 | 0.9784 | 0.9470 |
| 58.0 | 7.714 | 0.9801 | 0.9520 |
| 60.0 | 7.980 | 0.9815 | 0.9564 |
| 62.0 | 8.246 | 0.9828 | 0.9601 |
| 64.0 | 8.512 | 0.9840 | 0.9634 |
| 66.0 | 8.778 | 0.9850 | 0.9663 |
| 68.0 | 9.044 | 0.9859 | 0.9689 |
| 70.0 | 9.310 | 0.9867 | 0.9711 |
| 72.0 | 9.576 | 0.9874 | 0.9731 |
| 74.0 | 9.842 | 0.9880 | 0.9749 |
| 76.0 | 10.108 | 0.9886 | 0.9765 |
| 78.0 | 10.374 | 0.9891 | 0.9779 |
| 80.0 | 10.640 | 0.9896 | 0.9792 |
| 82.0 | 10.906 | 0.9901 | 0.9804 |
| 84.0 | 11.172 | 0.9905 | 0.9814 |
| 86.0 | 11.438 | 0.9908 | 0.9824 |
| 88.0 | 11.704 | 0.9912 | 0.9832 |
| 90.0 | 11.970 | 0.9915 | 0.9840 |
| 92.0 | 12.236 | 0.9918 | 0.9847 |
| 94.0 | 12.502 | 0.9921 | 0.9854 |
| 96.0 | 12.768 | 0.9923 | 0.9860 |
| 98.0 | 13.034 | 0.9926 | 0.9866 |
| 100.0 | 13.300 | 0.9928 | 0.9871 |

**Tab C.** Tabulated values for oxygen saturation (SaO_2_) at partial pressures of oxygen (pO_2_) between 0 and 100 Torr ( 0 to 13.3 kPa) at pH 7.2 and pH 7.4 for the white rhinoceros from interpolation based on eq.1+ eq.2.
