# Supplementary figures and images for: Odd haemoglobins in odd-toed ungulates: Impact of selected haemoglobin characteristics of the white rhinoceros (Ceratotherium simum) on the monitoring of the arterial oxygen saturation of haemoglobin
Source: PLoS One. 2019 Dec 30;14(12):e0226851. doi: 10.1371/journal.pone.0226851 (PMC6936770; doi:10.1371/journal.pone.0226851)

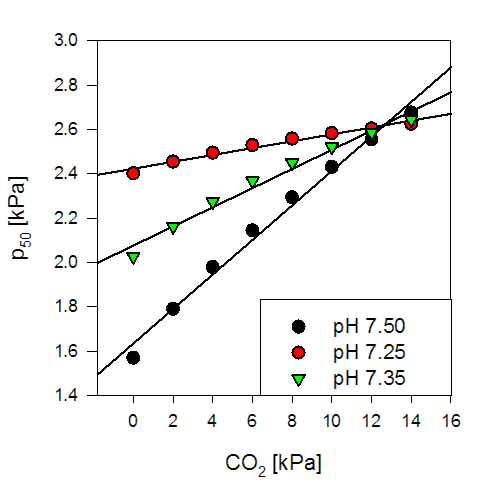

Supplement: S1 Fig — The corresponding p50 values show a decreasing impact of pCO2 at decreasing pH-values, in agreement with the results reported by Baumann et al. (TIF) [file pone.0226851.s002.TIF]

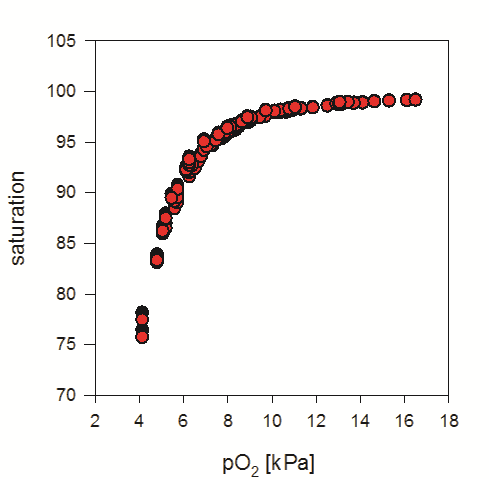

Supplement: S2 Fig — (TIF) [file pone.0226851.s003.TIF]

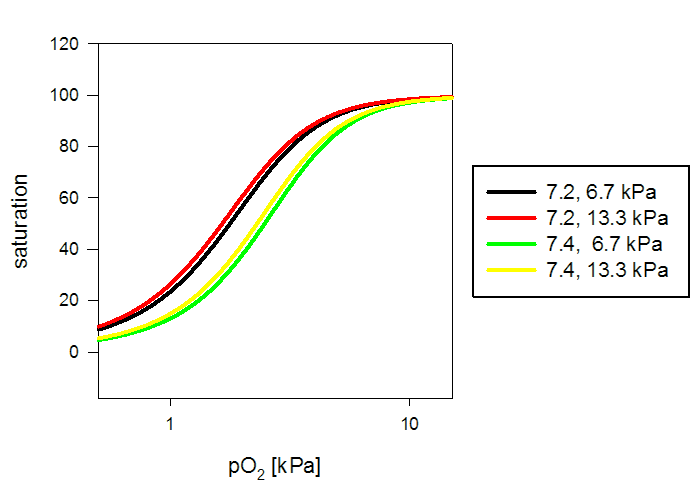

Supplement: S3 Fig — Note that an increase in pCO2 and a decrease in pH leads to a left-shift of the ODC, in contrast to experimental data by Baumann and colleagues. In contrast, the function represented by eq.2 and eq.3 reflect the experimentally observed shifts, since the experimental data were used to generate the equations. (TIF) [file pone.0226851.s004.TIF]
